# Effect of Ziltivekimab on Determinants of Hemoglobin in Patients with CKD Stage 3–5: An Analysis of a Randomized Trial (RESCUE)

**DOI:** 10.1681/ASN.0000000000000245

**Published:** 2023-12-13

**Authors:** Pablo E. Pergola, Michael Davidson, Camilla Jensen, Amir A. Mohseni Zonoozi, Dominic S. Raj, Philip Andreas Schytz, Katherine R. Tuttle, Vlado Perkovic

**Affiliations:** 1 Renal Associates P.A., San Antonio, Texas; 2 Section of Cardiology, Department of Medicine, University of Chicago, Chicago, Illinois; 3 Novo Nordisk A/S, Søborg, Denmark; 4 Division of Kidney Diseases and Hypertension, George Washington University School of Medicine, Washington, DC; 5 School of Medicine, University of Washington, and Providence Health Care, Spokane, Washington; 6 University of New South Wales Sydney, Sydney, Australia

**Keywords:** anemia, chronic inflammation, CKD, clinical trial

## Abstract

**Significance Statement:**

Systemic inflammation in CKD can lead to anemia. Ziltivekimab, a fully human monoclonal antibody targeting the IL-6 ligand, has been shown to reduce systemic inflammation in patients with CKD. It has also been shown to increase serum albumin in patients on hemodialysis with inflammation and hyporesponsiveness to treatment with erythropoiesis-stimulating agents. This exploratory analysis of the RESCUE clinical trial found that among patients with CKD stage 3–5 and systemic inflammation, ziltivekimab treatment significantly increased hemoglobin (Hb) levels after 12 weeks compared with placebo. Ziltivekimab was also associated with significant increases in serum iron levels, total iron-binding capacity, and transferrin saturation. No major safety concerns were reported. Further clinical trials are warranted to study ziltivekimab's potential for anemia management in patients with CKD.

**Background:**

In the phase 2 RESCUE clinical trial, ziltivekimab, a fully human monoclonal antibody against the IL-6 ligand, significantly reduced the biomarkers of inflammation compared with placebo, in patients with CKD and systemic inflammation (high-sensitivity C-reactive protein ≥2 mg/L). The aim of this subanalysis of RESCUE trial data was to assess the effect of ziltivekimab on Hb and iron homeostasis in this patient population.

**Methods:**

This was an analysis of exploratory end points from the RESCUE trial (NCT03926117), which included 264 adults with CKD stage 3–5 and high-sensitivity C-reactive protein ≥2 mg/L. Participants received placebo or subcutaneous ziltivekimab (7.5, 15, or 30 mg) (1:1:1:1) once every 4 weeks, up to 24 weeks. End points for this analysis were changes in Hb and biomarkers of iron homeostasis from baseline to week 12.

**Results:**

The trial was terminated early due to the coronavirus disease 2019 pandemic, and thus, data up to week 12 are presented. Hb levels significantly increased from baseline to week 12 with ziltivekimab 7.5, 15, and 30 mg (treatment differences versus placebo: +0.57 g/dl [95% confidence interval, 0.27 to 0.86], +1.05 g/dl [0.76 to 1.33], and +0.99 g/dl [0.70 to 1.28], respectively, all *P* < 0.001). Ziltivekimab was associated with significant increases in serum iron levels, total iron-binding capacity, and transferrin saturation from baseline to week 12 (*P* < 0.05 versus placebo for all doses and comparisons). Cases of sustained thrombocytopenia, sustained neutropenia, anemia, and iron deficiency anemia were infrequent and similar across all groups.

**Conclusions:**

Anti-inflammatory therapy with ziltivekimab improved the markers of anemia and iron homeostasis in people with stage 3–5 CKD and systemic inflammation, suggesting a possible role in anemia management.

## Visual Abstract

**Figure:**
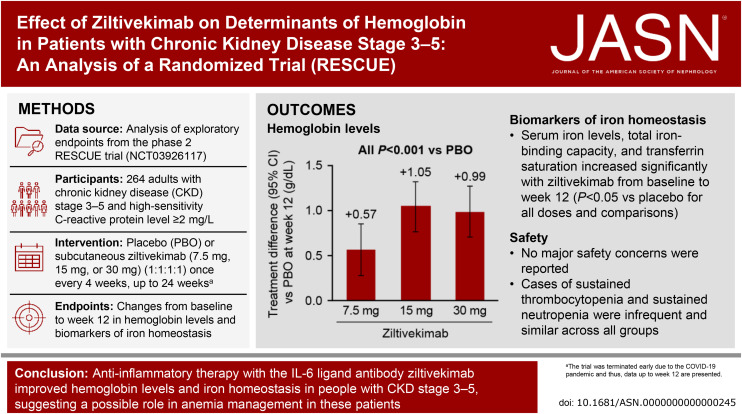

## Introduction

Anemia occurs in approximately 15% of patients with CKD^1^ and is associated with increased morbidity and mortality, as well as reduced quality of life.^2,3^ Between 30% and 50% of patients with kidney failure treated by chronic dialysis have high levels of biomarkers of inflammation, such as IL-6 and high-sensitivity C-reactive protein (hsCRP).^3^ Elevated levels of hsCRP are also associated with the onset of anemia in patients with earlier stages of CKD,^4^ and there is evidence that a reduction in inflammation, reflected by a decline in hsCRP, may improve outcomes in patients with CKD.^3^

Systemic inflammation can lead to dysregulation of iron homeostasis *via* upregulation of proinflammatory cytokines and acute phase reactants, including ferritin and hepcidin.^5,6^ IL-6 is a key proinflammatory cytokine that can increase hepcidin levels by increasing its transcription in hepatocytes *via* the Janus kinase 2 signal transducer and activator of transcription 3 pathway.^6^ The increase in hepcidin levels may lead to functional iron deficiency and anemia in patients with CKD.^7,8^ Furthermore, increased levels of IL-6 can suppress erythropoietin production and responsiveness, causing reduced erythropoiesis.^8,9^ Elevated levels of IL-6 are also an independent predictor of mortality in patients with kidney failure treated by hemodialysis.^10–14^ Importantly, inhibition of IL-6 decreases hsCRP levels in patients with CKD.^8,15^ As such, inhibition of IL-6 in patients with CKD may provide a potential treatment approach for anemia in this patient population. Current treatment approaches to anemia in patients with CKD, such as iron supplements and/or erythropoiesis-stimulating agents (ESAs), do not address the underlying causes of anemia, such as inflammation.^2,16,17^

Ziltivekimab is a fully human monoclonal antibody directed against the IL-6 ligand. In the randomized, double-blind, placebo-controlled phase 2 trial RESCUE (NCT03926117), ziltivekimab dose-dependently reduced the biomarkers of inflammation compared with placebo in patients with CKD stage 3–5 and systemic inflammation, defined as hsCRP ≥2 mg/L.^15^ In a phase 1/2 randomized, double-blind, placebo-controlled trial (NCT02868229), ziltivekimab significantly reduced inflammatory biomarkers and ESA requirements and increased serum albumin in patients treated by dialysis with inflammation and hyporesponsiveness to ESA therapy.^8^ However, the effect of ziltivekimab on hemoglobin (Hb) and iron homeostasis in a broader population of patients with CKD has not been studied. The aim of this study was to assess the effect of ziltivekimab on Hb and iron biomarkers in patients with CKD stage 3–5 and hsCRP ≥2 mg/L, using data from the RESCUE trial.

## Methods

### Patients

The RESCUE trial was a randomized, double-blind, placebo-controlled phase 2 trial conducted in the United States.^15^ Patients aged 18 years or older were eligible if they had an eGFR >10 and <60 ml/min per 1.73 m^2^ using the Chronic Kidney Disease Epidemiology Collaboration 2009 creatinine equation^18^ and systemic inflammation, which was defined as an hsCRP level of 2 mg/L or greater. Patients with an absolute neutrophil count of <2.0×10⁹ per L, a platelet count of <120×10⁹ per L, and a spot urine protein-to-creatinine ratio >4.0 g/g were excluded. Those who tested positive for active tuberculosis, HIV, or hepatitis B or C, as well as those with active infections or chronic use of immunosuppressive therapies, were also excluded. Patients were randomly assigned (1:1:1:1) to receive placebo or subcutaneous ziltivekimab at doses of 7.5, 15, or 30 mg once every 4 weeks, up to 24 weeks. Randomization was stratified by baseline Hb levels (<11 and ≥11 g/dl) and CKD stage (stages 3 and 4/5). This study was reviewed and approved by institutional review boards at the study sites. Written informed consent was obtained from each participant before study participation. All research activities adhered to the principles of the Declaration of Helsinki.

### Study Outcomes

Exploratory outcomes presented here were changes from baseline to week 12 in levels of Hb, iron, total iron-binding capacity (TIBC), transferrin saturation, reticulocyte Hb, ferritin, and hepcidin. A *post hoc* subgroup analysis was performed for levels of Hb, stratified by the baseline Hb level (<11 and ≥11 g/dl).

Safety parameters included anemia as an adverse event, neutropenia, and thrombocytopenia. Full information on safety parameters assessed during the RESCUE trial have been published previously.^15^

### Statistical Analysis

For Hb, change from baseline to week 12 was analyzed in the intention-to-treat population using a mixed model for repeated measures (MMRM) with baseline Hb (<11 and ≥11 g/dl), CKD stage (3a and 3b–5), concomitant iron medication, treatment group, visit, and treatment group-by-visit as fixed factors and baseline value as covariate. For baseline Hb subgroups (Hb <11 and Hb ≥11 g/dl), the model included CKD stage (3a and 3b–5), concomitant iron medication, treatment group, visit, and treatment group-by-visit-by-subgroup as fixed factors and baseline values as covariate. The MMRM model used an unstructured covariance matrix. Changes from baseline to weeks 1, 3, 4, 6, 8, and 12 were included in the MMRM; however, only changes from baseline to week 12 are presented. Missing values are assumed missing at random in the MMRM model. As the remaining parameters were only measured at baseline and week 12, change from baseline to week 12 was analyzed using an analysis of covariance model, including the same fixed factors and covariate as the models above for Hb. The analyses were not adjusted for multiplicity. *Post hoc* transferrin saturation quartile analyses used descriptive statistics (median and interquartile range) and were based on observed data.

SAS software (version 9.3 or later) proc mixed lsmestimate was used to obtain estimates and *P* values (F-test), applying the KENWARDROGER method to calculate the degrees of freedom.

## Results

### Baseline Characteristics

**Figure 1:**
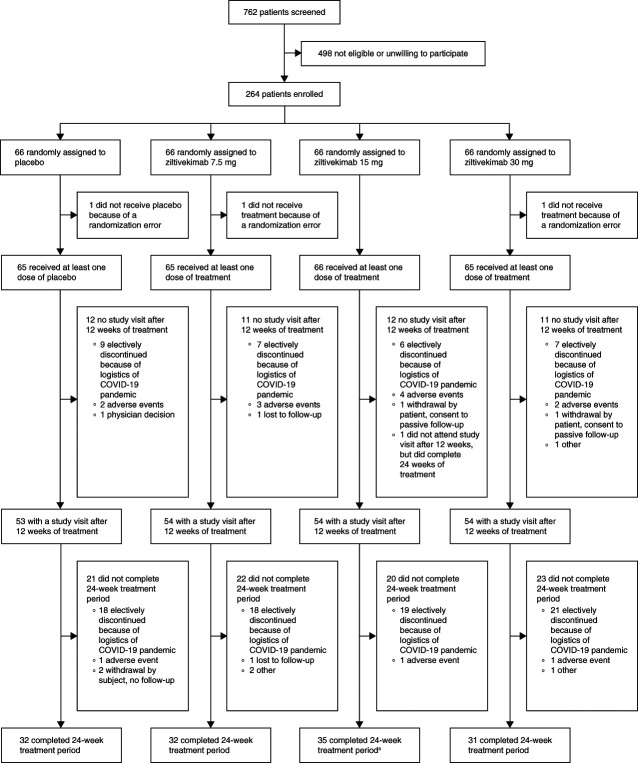
**RESCUE CONSORT diagram.** ^a^One participant did not attend a visit after 12 weeks but did complete the 24 weeks of treatment. “No follow-up” is defined as participants who withdrew consent and refused all further follow-up. “Passive follow-up” is defined as participants who withdrew consent and did not participate further with the trial procedures but consented to some follow-up. “Randomization error” is defined as participants who were randomized in error because they violated inclusion/exclusion criteria; these patients should not have been randomized and did not receive any treatment. COVID-19, coronavirus disease 2019. This figure was first published in Ridker PM *et al.* IL-6 inhibition with ziltivekimab in patients at high atherosclerotic risk (RESCUE): a double-blind, randomized, placebo-controlled, phase 2 trial. *Lancet*. 2021;397:2060–9, copyright Elsevier (2021), and reproduced with permission.

The trial enrolled 264 participants (June 17, 2019, to January 14, 2020), with 66 randomly assigned to each of the four treatment groups (ziltivekimab 7.5, 15, or 30 mg or placebo) (Figure 1). One patient each in the placebo, ziltivekimab 7.5 mg, and ziltivekimab 30 mg groups did not start treatment and were not included in the analyses.

With the onset of the coronavirus disease 2019 pandemic and concern that an exogenous cause of C-reactive protein increase in the general population could skew outcomes for the trial's primary inflammation end points, a decision was made by the executive committee and the sponsor to terminate the trial. This decision was made without knowledge of any outcome data in the trial, which was fully masked at that time, by the investigators, sponsor, or any study personnel.

The total number of patients with measurements contributing to each analysis is shown per group in the respective tables.

The mean age of participants who underwent randomization in the RESCUE trial was 66.4 years, and the mean Hb ranged between 12.2 and 12.5 g/dl across treatment groups; 129 participants (48.9%) were women. Seventy-seven patients (29.2%) had CKD stage 3a, 108 (40.9%) had stage 3b, 60 (22.7%) had stage 4, and 15 (5.7%) had stage 5. Baseline characteristics were similar across treatment groups (Table 1); 66 patients were receiving concomitant iron medication (20 [30.3%], 11 [16.7%], 16 [24.2%], and 19 [28.8%] in the placebo and ziltivekimab 7.5, 15, and 30 mg treatment groups, respectively); no patient was registered as receiving ESA treatment. Compared with patients with baseline Hb ≥11.0 g/dl, a higher proportion of patients with baseline Hb <11.0 g/dl were women (61.1% versus 45.7%) and had diabetes (83.3% versus 67.6%) (Supplemental Table 1). The group with baseline Hb <11.0 g/dl also had higher median baseline levels of hsCRP (7.1 versus 5.5 mg/L), mean baseline ferritin levels (210.3 versus 173.9 *µ*g/L) and hepcidin levels (116.9 versus 73.3 *µ*g/L), and lower mean baseline levels of serum iron (57.8 versus 72.1 *µ*g/dl), transferrin saturation (17.5% versus 20.9%), and mean eGFR (29.8 versus 38.7 ml/min per 1.73 m^2^) (Supplemental Table 1).

**Table 1:** Baseline Demographics and Clinical Characteristics of the RESCUE Trial Population

Baseline Characteristic	Placebo (*N*=66)	Ziltivekimab 7.5 mg (*N*=66)	Ziltivekimab 15 mg (*N*=66)	Ziltivekimab 30 mg (*N*=66)
Participants treated, *n*	65	65	66	65
Age, mean, yr	65.4	67.2	65.9	67.1
Women, *n* (%)	29 (43.9)	32 (48.5)	36 (54.5)	32 (48.5)
Race, *n* (%)				
White	50 (75.8)	48 (72.7)	49 (74.2)	52 (78.8)
Black or African American	16 (24.2)	18 (27.3)	12 (18.2)	14 (21.2)
Other	0 (0.0)	0 (0.0)	5 (7.6)	0 (0.0)
Hb, mean (SD), g/dl	12.4 (1.7)	12.5 (1.6)	12.5 (1.7)	12.2 (1.6)
Ferritin, mean (SD), *µ*g/L	168.5 (247.6)	182.3 (154.4)	166.2 (169.2)	208.4 (261.7)
Hepcidin, mean (SD), *µ*g/L	88.8 (89.9)	83.6 (68.2)	77.4 (54.5)	78.7 (56.2)
Iron, mean (SD), *µ*g/dl	69.4 (25.5)	69.7 (19.2)	67.7 (21.7)	70.0 (22.5)
TIBC, mean (SD), *µ*g/dl	332.3 (60.1)	319.2 (51.5)	332.7 (54.9)	329.4 (48.0)
Transferrin saturation, mean (SD), %	20.2 (8.6)	21.0 (6.8)	19.5 (7.3)	20.2 (6.4)
Reticulocyte Hb, mean (SD), pg	30.7 (1.7)	30.9 (1.6)	30.5 (1.8)	30.8 (2.4)
hsCRP, median (IQR), mg/L	5.8 (3.3–9.9)	5.5 (3.5–9.3)	5.7 (3.5–8.1)	5.8 (3.7–8.9)
GFR, mean (SD), ml/min per 1.73 m^2^	36.7 (13.8)	35.2 (12.0)	38.5 (12.6)	37.0 (12.1)
CKD stage, *n* (%)^a^				
3a	19 (28.8)	16 (24.2)	23 (34.8)	19 (28.8)
3b	23 (34.8)	30 (45.5)	29 (43.9)	26 (39.4)
4	17 (25.8)	16 (24.2)	10 (15.2)	17 (25.8)
5	5 (7.6)	3 (4.5)	4 (6.1)	3 (4.5)
Diabetes, *n* (%)^b^	50 (75.8)	41 (62.1)	48 (72.7)	48 (72.7)
ASCVD, *n* (%)	37 (56.1)	29 (43.9)	27 (40.9)	33 (50.0)
Statin use, *n* (%)	45 (68.2)	44 (66.7)	45 (68.2)	45 (68.2)
Concomitant iron medication	20 (30.3)	11 (16.7)	16 (24.2)	19 (28.8)

Data from all 264 patients randomized in the RESCUE trial. ASCVD, atherosclerotic cardiovascular disease; Hb, hemoglobin; HbA_1c_, glycosylated hemoglobin; hsCRP, high-sensitivity C-reactive protein; IQR, interquartile range; TIBC, total iron-binding capacity.

a Baseline CKD values were based on laboratory analyses and calculated as the average of all eGFR assessments before the first dose. CKD stages 3a and 3b indicate patients with stage 3 CKD disease with respective baseline eGFR rates of 45–59 and 30–44 ml/min per 1.73 m^2^.

b Includes patients with HbA_1c_ >6.5% and those with a history of diabetes at baseline or patients on diabetes medication at baseline. Diabetes history of patients was identified using the Medical Dictionary for Regulatory Activities (MedDRA) version 22.0.

### Effects of Ziltivekimab on Hb Levels

Ziltivekimab was associated with a significant increase in Hb levels from baseline to week 12 versus placebo. The mean changes in Hb from baseline to week 12 were −0.22 g/dl for placebo and 0.34, 0.82, and 0.77 g/dl for ziltivekimab 7.5, 15, and 30 mg, respectively, corresponding to treatment differences versus placebo of 0.57 (95% confidence interval, 0.27 to 0.86, *P* < 0.001), 1.05 (0.76 to 1.33, *P* < 0.001), and 0.99 (0.70 to 1.28, *P* < 0.001), respectively (Table 2).

**Table 2:** Hb Levels at Baseline and at Week 12 and Estimated Change From Baseline and Treatment Difference

Hb Level	Placebo	Ziltivekimab 7.5 mg	Ziltivekimab 15 mg	Ziltivekimab 30 mg
Overall population				
Hb at baseline, mean (SD), g/dl	12.35 (1.71)	12.50 (1.63)	12.52 (1.70)	12.21 (1.55)
Hb at week 12, observed mean (SD), g/dl	12.20 (1.87)	12.67 (1.91)	13.39 (1.69)	13.05 (1.54)
Change from baseline to week 12, g/dl, estimated mean^a^ (95% CI) [*n*]	−0.22 (−0.44 to −0.01) [*57*]	0.34 (0.12 to 0.56) [*53*]	0.82 (0.61 to 1.03) [*60*]	0.77 (0.55 to 0.99) [*57*]
Treatment difference^a^ (95% CI), g/dl	—	0.57 (0.27 to 0.86)^c^	1.05 (0.76 to 1.33)^c^	0.99 (0.70 to 1.28)^c^
Baseline Hb <11 g/dl				
Hb at baseline, mean (SD), g/dl	9.92 (0.85)	10.23 (0.58)	10.54 (0.67)	10.05 (0.96)
Hb at week 12, observed mean (SD), g/dl	9.72 (1.32)	10.38 (0.86)	11.50 (1.01)	11.25 (1.13)
Change from baseline to week 12, g/dl, estimated mean^b^ (95% CI) [*n*]	−0.29 (−0.75 to 0.17) [*12*]	0.07 (−0.38 to 0.51) [*13*]	0.80 (0.33 to 1.27) [*11*]	1.18 (0.71 to 1.66) [*11*]
Treatment difference^b^ (95% CI), g/dl	—	0.36 (–0.26 to 0.98)	1.09 (0.45 to 1.73)^c^	1.48 (0.83 to 2.12)^c^
Baseline Hb ≥11 g/dl				
Hb at baseline, mean (SD), g/dl	12.94 (1.29)	13.05 (1.28)	13.05 (1.48)	12.79 (1.09)
Hb at week 12, observed mean (SD), g/dl	12.86 (1.37)	13.42 (1.52)	13.82 (1.51)	13.48 (1.29)
Change from baseline to week 12, g/dl, mean (95% CI)^b^ [*n*]	−0.22 (−0.45 to 0.01) [*45*]	0.41 (0.16 to 0.65) [*40*]	0.81 (0.58 to 1.04) [*49*]	0.65 (0.42 to 0.89) [*46*]
Treatment difference^b^ (95% CI), g/dl	—	0.62 (0.29 to 0.96)^c^	1.03 (0.71 to 1.35)^c^	0.87 (0.55 to 1.20)^c^

CI, confidence interval; Hb, hemoglobin; *n*, number of participants with baseline and week 12 Hb values.

a Estimated from a mixed model for repeated measures, with baseline Hb (<11 and ≥11 g/dl), CKD stage (3a and 3b–5), concomitant iron medication, treatment group, visit, and treatment group-by-visit as fixed factors and baseline value as covariate.

b Estimated from a mixed model for repeated measures, with CKD stage (3a and 3b–5), concomitant iron medication, treatment group, visit, and treatment group-by-visit-by-subgroup as fixed factors and baseline values as covariate.

c *P* < 0.001 versus placebo.

**Figure 2:**
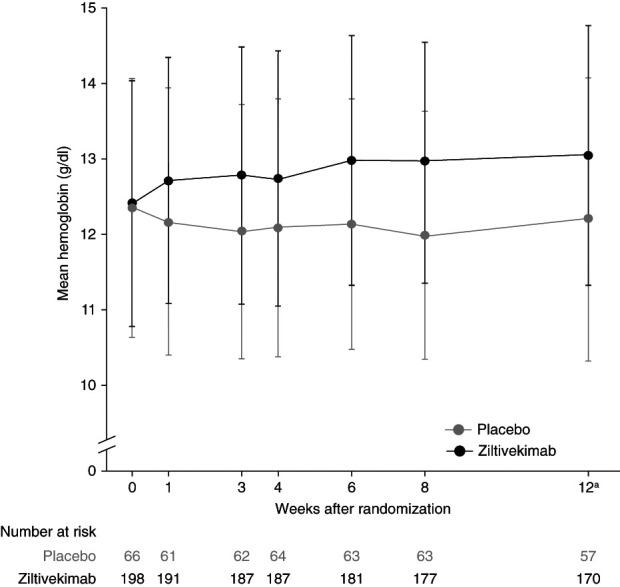
**Mean Hb levels for the pooled ziltivekimab groups versus placebo over time (intention-to-treat population).** ^a^Prespecified time point. Data are presented as mean values and SDs (error bars). Ziltivekimab data include the pooled 7.5, 15, and 30 mg doses; *n* values for the placebo and ziltivekimab 7.5, 15, and 30 mg treatment groups at week 12 in the overall population were 57, 53, 60, and 57 participants. *n* values shown below the graph are for participants contributing to the analysis at the time point indicated. Hb, hemoglobin.

Similar results were noted in subgroups stratified by baseline Hb <11 or ≥11 g/dl (Table 2). In an analysis using pooled data from all participants receiving ziltivekimab, ziltivekimab treatment was associated with a rapid and sustained increase in Hb levels over 12 weeks; differences between ziltivekimab and placebo groups could be observed as early as week 1 (Figure 2).

### Effects of Ziltivekimab on Biomarkers of Iron Homeostasis

Ziltivekimab was associated with a statistically significant increase in serum iron levels, TIBC, and transferrin saturation compared with placebo across all doses (Table 3). Reticulocyte Hb levels increased significantly in the ziltivekimab 15 and 30 mg treatment groups. No significant changes were observed for ferritin or hepcidin in the ziltivekimab treatment groups.

**Table 3:** Change From Baseline to Week 12 for Additional Biomarkers of Anemia

Biomarker	Outcome	Placebo	Ziltivekimab 7.5 mg	Ziltivekimab 15 mg	Ziltivekimab 30 mg
Ferritin	Change from baseline to week 12 *µ*g/L, estimated mean^a^ (95% CI) [*n*]	–16.56 (–45.10 to 11.97) [*57*]	–23.77 (–52.59 to 5.04) [*58*]	–7.13 (–35.44 to 21.18) [*61*]	–22.38 (–51.74 to 6.99) [*57*]
	Treatment difference^a^ (95% CI), *µ*g/L	—	–7.21 (–43.61 to 29.18)	9.43 (–26.70 to 45.56)	–5.81 (–42.65 to 31.02)
Hepcidin	Change from baseline to week 12, *µ*g/L, estimated mean^a^ (95% CI) [*n*]	–3.85 (–16.63 to 8.93) [*56*]	–13.49 (–26.48 to –0.49) [*57*]	–15.50 (–28.21 to –2.78) [*60*]	–18.79 (–31.98 to –5.61) [*56*]
	Treatment difference^a^ (95% CI), *µ*g/L	—	–9.64 (–25.97 to 6.69)	–11.65 (–27.84 to 4.55)	–14.94 (–31.45 to 1.56)
Iron	Change from baseline to week 12, *µ*g/dl, estimated mean^a^ (95% CI) [*n*]	–1.91 (–10.94 to 7.13) [*57*]	16.34 (7.24 to 25.43) [*58*]	28.82 (19.84 to 37.81) [*61*]	32.89 (23.61 to 42.17) [*57*]
	Treatment difference^a^ (95% CI), *µ*g/dl	—	18.24 (6.75 to 29.74)^c^	30.73 (19.33 to 42.13)^d^	34.79 (23.18 to 46.40)^d^
TIBC	Change from baseline to week 12, *µ*g/dl, estimated mean^a^ (95% CI) [*n*]	–0.04 (–8.04 to 7.96) [*56*]	14.23 (6.21 to 22.25) [*58*]	17.72 (9.86 to 25.59) [*61*]	24.36 (16.20 to 32.51) [*57*]
	Treatment difference^a^ (95% CI), *µ*g/dl	—	14.27 (4.09 to 24.44)^c^	17.76 (7.68 to 27.84)^d^	24.39 (14.12 to 34.66)^d^
Transferrin saturation	Change from baseline to week 12, %, estimated mean^a^ (95% CI) [*n*]	–0.26 (–2.80 to 2.27) [*57*]	3.77 (1.21 to 6.32) [*58*]	7.05 (4.53 to 9.57) [*61*]	7.69 (5.08 to 10.30) [*57*]
	Treatment difference^a^ (95% CI), %	—	4.03 (0.80 to 7.27)^b^	7.31 (4.12 to 10.51)^d^	7.95 (4.70 to 11.21)^d^
Reticulocyte hemoglobin	Change from baseline to week 12, pg, estimated mean^a^ (95% CI) [*n*]	0.19 (–0.17 to 0.54) [*55*]	0.60 (0.23 to 0.97) [*52*]	0.75 (0.38 to 1.11) [*58*]	0.87 (0.51 to 1.24) [*55*]
	Treatment difference^a^ (95% CI), pg	—	0.41 (–0.05 to 0.87)	0.56 (0.10 to 1.01)^b^	0.69 (0.23 to 1.14)^c^

CI, confidence interval; Hb, hemoglobin; *n*, number of participants with baseline and week 12 values; TIBC, total iron-binding capacity.

a Estimated from an analysis of covariance with baseline Hb (<11 and ≥11 g/dl), CKD stage (3a and 3b–5), concomitant iron medication, treatment group, visit, and treatment group-by-visit as fixed factors and baseline value as covariate.

b *P* < 0.05 versus placebo.

c *P* < 0.01 versus placebo.

d *P* < 0.001 versus placebo.

Data on changes in levels of anemia biomarkers, stratified by Hb levels at baseline, are presented in Supplemental Table 2. Overall, these subgroup data aligned with the overall population, with significant increases in serum iron levels and TIBC compared with placebo in both the <11 and ≥11 g/dl subgroups across most ziltivekimab doses. A significant increase in reticulocyte levels was observed in the baseline Hb ≥11 g/dl subgroup for the 15 and 30 mg treatment groups. For transferrin saturation, changes versus placebo were significant for the subgroups with baseline Hb ≥11 g/dl, but not for subgroups with baseline Hb <11 g/dl, with the exception of the ziltivekimab 15 mg group.

When transferrin saturation was analyzed by baseline transferrin saturation quartiles (quartile 1: ≤15.5%, quartile 2: >15.5% and ≤19.0%, quartile 3: >19.0% and ≤23.5%, and quartile 4: >23.5%), transferrin saturation was similar for the corresponding placebo and pooled ziltivekimab quartile groups at baseline (Supplemental Table 3). At week 12, transferrin saturation decreased across most quartiles with placebo (with the exception of quartile 1), but increased markedly in all quartiles for ziltivekimab, including in higher quartiles of transferrin saturation at baseline. In an analogous quartile analysis of baseline hsCRP levels by baseline transferrin saturation, there was an inversely proportional relationship between transferrin saturation quartiles and hsCRP (Supplemental Table 3).

### Adverse Events

Ziltivekimab was well tolerated, with no major safety concerns reported (Table 4).^15^ The numbers of cases of AKI, anemia, and iron deficiency anemia were low and similar across treatment groups. One case of sustained grade 2 neutropenia was reported in the ziltivekimab 7.5 mg group; there were no cases of grade 3 or 4 neutropenia in any treatment arm. No cases of grade 2–4 sustained thrombocytopenia were reported.

**Table 4:** RESCUE Trial Safety Outcomes Relevant to Anemia and CKD

Safety Outcome	Placebo (*N*=65)	Ziltivekimab 7.5 mg (*N*=65)	Ziltivekimab 15 mg (*N*=66)	Ziltivekimab 30 mg (*N*=65)
Any TEAEs, n (%)	45 (69.2)	43 (66.2)	44 (66.7)	47 (72.3)
Mild	19 (29.2)	15 (23.1)	18 (27.3)	16 (24.6)
Moderate	18 (27.7)	16 (24.6)	19 (28.8)	23 (35.4)
Severe	8 (12.3)	12 (18.5)	7 (10.6)	8 (12.3)
System organ class preferred term				
AKI, *n* (%)	1 (1.5)	2 (3.1)	0 (0.0)	2 (3.1)
Anemia, *n* (%)	3 (4.6)	1 (1.5)	1 (1.5)	1 (1.5)
Iron deficiency anemia, *n* (%)	2 (3.1)	2 (3.1)	0 (0.0)	0 (0.0)
	**(*N*=65)**	**(*N*=65)**	**(*N*=66)**	**(*N*=64)**
Sustained^a^ neutropenia^b^, *n* (%)				
Grade 1 (<2000–1500 cells/mm^3^)	1 (1.5)	1 (1.5)	2 (3.0)	1 (1.6)
Grade 2 (<1500–1000 cells/mm^3^)	0 (0.0)	1 (1.5)	0 (0.0)	0 (0.0)
Grade 3 or 4 (<1000 cells/mm^3^)	0 (0.0)	0 (0.0)	0 (0.0)	0 (0.0)
Sustained^a^ thrombocytopenia^b^, *n* (%)				
Grade 1 (<100,000–75,000 cells/mm^3^)	0 (0.0)	0 (0.0)	2 (3.0)	1 (1.6)
Grade 2, 3, or 4 (<75,000 cells/mm^3^)	0 (0.0)	0 (0.0)	0 (0.0)	0 (0.0)

AE, adverse event; CTCAE, Common Terminology Criteria for Adverse Events; *n*, number of patients with adverse event; TEAE, treatment-emergent adverse event.

a “Sustained” was defined as having the condition in at least two consecutive visits.

b By CTCAE grade. %=100×*n*/*N*, where *N*=the number of patients with at least two nonmissing values after the first dose of the study drug. CTCAE Grade is assigned as the grade from the second value. TEAEs are defined as AEs that began or worsened on or after the date of the first dose of the study drug up to the end of the safety follow-up. One participant in the ziltivekimab 15 mg group reported short-term discomfort at the injection site.

## Discussion

Ziltivekimab was associated with significantly increased levels of Hb from baseline to week 12 versus placebo. The levels of biomarkers of iron metabolism (serum iron, TIBC, and transferrin saturation) also increased from baseline with ziltivekimab compared with placebo, suggesting a potential role for anti-inflammatory therapy in the treatment of anemia in CKD. Although hepcidin levels decreased over the 12-week treatment period, with greater reductions observed with higher ziltivekimab doses, changes were not statistically significant compared with the placebo group. Nevertheless, it can be postulated that these reductions were physiologically meaningful because the reduction in hepcidin levels would explain the observed effects on iron metabolism and ultimately on Hb levels.

The results of this analysis from the RESCUE trial in patients with CKD stage 3–5 are consistent with the results of the phase 1/2 study, which showed that ziltivekimab was associated with significant improvement in markers of inflammation, reduced ESA requirements, and increased serum albumin in patients on hemodialysis with inflammation and hyporesponsiveness to ESA therapy.^8^

Transferrin saturation may be a better marker of iron availability for erythropoiesis than ferritin in inflammatory conditions because it seems to be less susceptible to direct changes caused by inflammation than ferritin.^19^ This explanation is supported by the results from the quartile analysis by baseline transferrin saturation in our study, which indicated that ziltivekimab increased transferrin saturation, with greater relative increases in patients with low baseline transferrin saturation. Interestingly, there was an inverse proportional relationship between hsCRP levels and baseline transferrin saturation quartiles, suggesting that inflammation as measured by hsCRP may contribute to low bioavailable iron for hematopoiesis, and iron availability improved through reduced inflammation because of the use of ziltivekimab.

For outcomes stratified by baseline Hb levels, the results were generally similar for participants with Hb <11 g/dl and for those with Hb ≥11 g/dl. These thresholds do not take into account sex-specific differences in Hb levels, with men typically having higher levels than women,^20^ and, therefore, sex-specific thresholds may need to be used to better understand responses to treatment. A difference in baseline Hb levels between men and women was also noted in the RESCUE trial (Supplemental Figure 1 and Supplemental Table 4). It should also be noted that most patients had baseline Hb levels ≥11 g/dl, rather than <11 g/dl (*n*=207 versus *n*=53, respectively). Analyses stratified by sex and Hb levels could provide more detailed insights into the determinants of Hb in patients with CKD and anemia, but a larger sample needs to be studied to arrive at conclusions. The effects of ziltivekimab on ESA dosage in patients with non–dialysis-dependent CKD also remain to be determined because only one patient was registered as receiving ESA treatment at baseline. Given the positive effect of ziltivekimab on Hb levels, it is plausible that ESA requirements could decrease or stop, as was observed in the phase 1/2 study.^8^ The relatively short duration of the RESCUE trial (12 weeks), owing to early termination because of the coronavirus disease 2019 pandemic, was an additional limitation of the analyses.

In conclusion, in the phase 2 RESCUE trial, the IL-6 ligand antibody ziltivekimab significantly improved the levels of Hb, serum iron, total iron-binding capacity, and transferrin saturation versus placebo in patients with CKD stage 3–5. These findings suggest that ziltivekimab has the potential to improve anemia in patients with CKD stage 3–5, *via* the inhibition of IL-6. Together with the results from the phase 1/2 trial, the results of this analysis suggest a new therapeutic approach for treating anemia in patients with CKD.^8^ Treating inflammation, a significant underlying mechanism of disease in many patients with anemia in CKD, has the potential to increase Hb without the need for ESA or iron therapies.

The ongoing large-scale phase 3 cardiovascular outcomes trial ZEUS is investigating the effect of ziltivekimab (15 mg) compared with placebo in 6200 patients with stage 3–4 CKD and elevated hsCRP who are at high risk of atherosclerotic events.^21^ The trial includes change in Hb levels from baseline to year 2 as an exploratory end point and will provide further evidence regarding the use of ziltivekimab to improve anemia in patients with CKD stage 3–4. In addition, if a similar increase in Hb is noticed in the long term in patients with preexisting cardiovascular disease treated with ziltivekimab, it could be tested as a potential driver for improved cardiovascular outcomes in those patients.

## Supplementary Material

## Data Availability

Individual participant data will be shared in data sets in a deidentified and anonymized format, including data sets from Novo Nordisk–sponsored clinical research completed after 2001 for product indications approved in both the EU and the United States. The study protocol and redacted clinical study report will be available according to Novo Nordisk data sharing commitments. Data will be available permanently after research completion and approval of product and product use in the EU and the United States. Data will only be shared with *bona fide* researchers submitting a research proposal and requesting access to data, for use as approved by the independent review board and according to its charter. The access request proposal form and the access criteria can be found online at www.novonordisk-trials.com. Data will be made available on a specialized Statistical Analysis System data platform.

## Supplemental Material

Supplemental Table 1. Baseline demographics and clinical characteristics of the RESCUE trial population, stratified by baseline Hb.

Supplemental Table 2. Mean levels of additional biomarkers of anemia at baseline and week 12 stratified by baseline Hb.

Supplemental Table 3. Transferrin saturation at baseline and week 12 and CRP at baseline, by baseline transferring saturation quartile groups, for pooled ziltivekimab groups versus placebo.

Supplemental Table 4. Proportion of participants with baseline Hb <12/<13 g/dL or ≥12/≥13 g/dL by sex.

Supplemental Figure 1. Distribution of baseline Hb by sex.
