# Supplementary material for: Effect of Ziltivekimab on Determinants of Hemoglobin in Patients with CKD Stage 3–5: An Analysis of a Randomized Trial (RESCUE)
Source: J Am Soc Nephrol. 2023 Dec 13;35(1):74–84. doi: 10.1681/ASN.0000000000000245 (PMC10786611; doi:10.1681/ASN.0000000000000245)
Supplement: Supplementary file 1 [file jasn-35-074-s001.pdf]

Supplemental Material

Effect of Ziltivekimab on Determinants of Hemoglobin in Patients With Chronic Kidney Disease Stages 3–5: An Analysis of a Randomized Trial (RESCUE)

Pablo E. Pergola,[1] Michael Davidson,[2] Camilla Jensen,[3] Amir A. Mohseni Zonoozi,[3] Dominic S. Raj,[4] Philip Andreas Schytz,[3] Katherine R. Tuttle,[5] and Vlado Perkovic[6]

[1]Renal Associates P.A., San Antonio, Texas, USA; [2]Section of Cardiology, Department of Medicine, University of Chicago, Chicago, Illinois, USA; [3]Novo Nordisk A/S, Søborg, Denmark; [4]Division of Kidney Diseases and Hypertension, George Washington University School of Medicine, Washington, DC, USA; [5]School of Medicine, University of Washington, and Providence Health Care, Spokane, Washington, USA; [6]University of New South Wales Sydney, Sydney, Australia

Supplemental Table 1. Baseline Demographics and Clinical Characteristics of the RESCUE Trial Population, Stratified by Baseline Hb

	**Hb <11 g/dL (*n*=54)**	**Hb ≥11 g/dL (*n*=210)**
Age, mean, years	65.6	66.6
Women, *n* (%)	33 (61.1)	96 (45.7)
Race, *n* (%)		
White	37 (68.5)	162 (77.1)
Black or African American	17 (31.5)	43 (20.5)
Other	0 (0.0)	5 (2.4)
Diabetes, *n* (%)[a]	45 (83.3)	142 (67.6)
ASCVD, *n* (%)	26 (48.1)	100 (47.6)
Statin use, *n* (%)	40 (74.1)	139 (66.2)
Concomitant iron medication	25 (46.3)	41 (19.5)
hsCRP, median, mg/L	7.1	5.5
Ferritin, mean (SD), µg/L	210.3 (219.6)	173.9 (211.0)
Hepcidin, mean (SD), µg/L	116.9 (99.0)	73.3 (55.2)
Serum iron, mean (SD), µg/dL	57.8 (16.1)	72.1 (22.7)
Transferrin saturation, mean (SD), %	17.5 (5.6)	20.9 (7.5)
GFR, mean (SD), mL/min/1.73 m^2	29.8 (11.4)	38.7 (12.3)
CKD stage, *n* (%)[b]		
3a	7 (13.0)	70 (33.3)
3b	19 (35.2)	89 (42.4)
4	21 (38.9)	39 (18.6)

5	7 (13.0)	8 (3.8)

ASCVD, atherosclerotic cardiovascular disease; CKD, chronic kidney disease; eGFR, estimated glomerular filtration rate; hsCRP, high-sensitivity C-reactive protein; SD, standard deviation.

[a]Includes patients with HbA_{1c} >6.5% and those with a history of diabetes at baseline or patients on diabetes medication at baseline. Diabetes history of patients was identified using the Medical Dictionary for Regulatory Activities (MedDRA) Version 22.0. [b]Baseline CKD values based on laboratory analyses and calculated as the average of all eGFR assessments prior to the first dose. CKD stages 3a and 3b indicate patients with stage 3 CKD disease with respective baseline eGFR rates of 45–59 mL/min/1.73 m^2 and 30–44 mL/min/1.73 m^2.

Supplemental Table 2. Mean Levels of Additional Biomarkers of Anemia at Baseline and Week 12 Stratified by Baseline Hb

	**Baseline Hb <11 g/dL**				**Baseline Hb ≥11 g/dL**			
	**Placebo**	**Ziltivekimab 7.5 mg**	**Ziltivekimab 15 mg**	**Ziltivekimab 30 mg**	**Placebo**	**Ziltivekimab 7.5 mg**	**Ziltivekimab 15 mg**	**Ziltivekimab 30 mg**
Ferritin								
Ferritin at baseline, mean (SD), µg/L	280.4 (305.2)	261.6 (214.8)	123.5 (126.2)	184.3 (189.2)	141.0 (226.4)	162.8 (131.1)	177.7 (178.3)	214.9 (279.3)
Ferritin at week 12, observed mean (SD), µg/L	300.4 (277.3)	222.5 (202.5)	157.5 (170.9)	172.5 (169.7)	105.2 (112.6)	131.8 (145.1)	165.8 (205.7)	154.3 (191.0)
Change from baseline to week 12, estimated mean[a] (95% CI), µg/L, [*n*]	38.8 (–18.9 to 96.6) [*12*]	–19.0 (–74.4 to 36.5) [*13*]	6.3 (–50.9 to 63.5) [*12*]	–28.6 (–88.4 to 31.2) [*11*]	–43.8 (–74.2 to –13.3) [45]	–37.1 (–68.2 to –6.0) [*45*]	–23.1 (–53.2 to 7.0) [*49*]	–33.6 (–65.2 to –2.0) [*46*]
Treatment difference[a] (95% CI), µg/L	–	–57.8 (–135.7 to 20.1)	–32.5 (–112.9 to 47.8)	–67.4 (–148.9 to 14.0)	–	6.7 (–34.5 to 47.8)	20.7 (–19.8 to 61.2)	10.2 (–31.1 to 51.5)

	Baseline Hb <11 g/dL				Baseline Hb ≥11 g/dL			
	**Placebo**	**Ziltivekimab 7.5 mg**	**Ziltivekimab 15 mg**	**Ziltivekimab 30 mg**	**Placebo**	**Ziltivekimab 7.5 mg**	**Ziltivekimab 15 mg**	**Ziltivekimab 30 mg**
Hepcidin								
Hepcidin at baseline, mean (SD), µg/L	168.7 (146.7)	130.9 (99.3)	76.5 (46.9)	94.7 (56.2)	69.2 (56.0)	71.8 (53.1)	77.6 (56.8)	74.7 (56.0)
Hepcidin at week 12, observed mean (SD), µg/L	152.7 (111.1)	114.8 (115.5)	83.8 (53.5)	78.9 (41.8)	57.8 (55.3)	53.7 (55.2)	58.4 (50.8)	55.1 (58.4)
Change from baseline to week 12, estimated mean[a] (95% CI), µg/L, [*n*]	11.3 (–15.3 to 37.8) [*12*]	–8.2 (–34.2 to 17.9) [*12*]	–2.9 (–28.5 to 22.6) [*12*]	–16.4 (–43.1 to 10.4) [*11*]	–14.4 (–28.2 to –0.6) [*44*]	–21.4 (–35.5 to –7.4) [*45*]	–25.3 (–38.9 to –11.7) [*48*]	–26.1 (–40.4, –11.8) [*45*]
Treatment difference[a] (95% CI), µg/L	–	–19.4 (–55.0 to 16.2)	–14.2 (–50.6 to 22.3)	–27.6 (–64.5 to 9.3)	–	–7.0 (–25.5 to 11.5)	–10.9 (–29.1 to 7.4)	–11.7 (–30.3 to 7.0)

	Baseline Hb <11 g/dL				Baseline Hb ≥11 g/dL			
	**Placebo**	**Ziltivekimab 7.5 mg**	**Ziltivekimab 15 mg**	**Ziltivekimab 30 mg**	**Placebo**	**Ziltivekimab 7.5 mg**	**Ziltivekimab 15 mg**	**Ziltivekimab 30 mg**
Iron								
Iron at baseline, mean (SD), µg/dL	58.7 (23.1)	58.4 (15.7)	55.9 (12.2)	58.5 (13.4)	72.1 (25.6)	72.5 (19.1)	70.9 (22.6)	73.1 (23.5)
Iron at week 12, observed mean (SD), µg/dL	66.8 (36.5)	74.5 (29.7)	95.9 (42.8)	85.0 (14.6)	66.8 (32.5)	90.9 (30.8)	98.6 (38.3)	108.2 (41.9)
Change from baseline to week 12, estimated mean[a] (95% CI), µg/dL, [*n*]	4.7 (–13.6 to 22.3) [*12*]	14.4 (–3.2 to 32.0) [*13*]	35.3 (17.1 to 53.5) [*12*]	26.6 (7.5 to 45.7) [*11*]	–4.6 (–14.2 to 5.0) [*45*]	16.0 (6.1 to 25.9) [*45*]	26.3 (16.7 to 35.8) [*49*]	33.5 (23.4 to 43.5) [*46*]
Treatment difference[a] (95% CI), µg/dL	–	9.7 (–15.0 to 34.4)	30.6 (5.3 to 55.9)*	21.9 (–3.9 to 47.7)	–	20.6 (7.6 to 33.7)**	30.9 (18.2 to 43.7)***	38.1 (25.1 to 51.1)***

	Baseline Hb <11 g/dL				Baseline Hb ≥11 g/dL			
	**Placebo**	**Ziltivekimab 7.5 mg**	**Ziltivekimab 15 mg**	**Ziltivekimab 30 mg**	**Placebo**	**Ziltivekimab 7.5 mg**	**Ziltivekimab 15 mg**	**Ziltivekimab 30 mg**
TIBC								
TIBC at baseline, mean (SD), μg/dL	306.6 (63.4)	300.9 (41.2)	330.0 (74.3)	319.6 (56.0)	338.7 (58.2)	323.6 (53.0)	333.5 (49.3)	332.0 (45.8)
TIBC at week 12, observed mean (SD), μg/dL	294.6 (48.8)	312.5 (53.1)	342.2 (59.6)	331.8 (52.5)	342.5 (43.1)	344.9 (57.7)	354.0 (50.2)	363.1 (44.4)
Change from baseline to week 12, estimated mean[a] (95% CI), μg/dL, [*n*]	–9.9 (–26.0 to 6.3) [*12*]	11.2 (–4.3 26.8) [*13*]	13.1 (–2.9 to 29.1) [*12*]	20.7 (4.0 to 37.5) [*11*]	6.5 (–2.2 to 15.1) [*44*]	18.8 (10.1 to 27.5) [*45*]	22.8 (14.4 to 31.2) [*49*]	29.2 (20.3 to 38.1) [*46*]
Treatment difference[a] (95% CI), μg/dL	–	21.1 (−0.7 to 42.9)	23.0 (0.6 to 45.4)*	30.6 (7.9 to 53.4)**	–	12.4 (0.8 to 23.9)*	16.4 (5.0 to 27.7)**	22.8 (11.2 to 34.3)***

	Baseline Hb <11 g/dL				Baseline Hb ≥11 g/dL			
	**Placebo**	**Ziltivekimab 7.5 mg**	**Ziltivekimab 15 mg**	**Ziltivekimab 30 mg**	**Placebo**	**Ziltivekimab 7.5 mg**	**Ziltivekimab 15 mg**	**Ziltivekimab 30 mg**
Transferrin saturation								
Transferrin saturation at baseline, mean (SD), %	18.5 (8.3)	18.1 (5.0)	16.1 (4.3)	17.5 (4.3)	20.6 (8.7)	21.7 (7.0)	20.4 (7.7)	20.9 (6.7)
Transferrin saturation at week 12, observed mean (SD), %	21.4 (9.9)	23.2 (9.5)	26.8 (11.5)	24.6 (2.4)	18.6 (11.3)	25.1 (8.7)	26.8 (11.2)	28.4 (10.8)
Change from baseline to week 12, estimated mean[a] (95% CI), %, [*n*]	1.8 (–3.3 to 6.9) [*12*]	4.4 (–0.5 to 9.4) [*13*]	9.0 (3.9 to 14.1) [*12*]	6.6 (1.2 to 11.9) [*11*]	–1.5 (–4.2 to 1.2) [*45*]	2.9 (0.2 to 5.7) [*45*]	5.9 (3.2 to 8.6) [*49*]	7.3 (4.5, 10.1) [*46*]

	Baseline Hb <11 g/dL				Baseline Hb ≥11 g/dL			
	**Placebo**	**Ziltivekimab 7.5 mg**	**Ziltivekimab 15 mg**	**Ziltivekimab 30 mg**	**Placebo**	**Ziltivekimab 7.5 mg**	**Ziltivekimab 15 mg**	**Ziltivekimab 30 mg**
Treatment difference[a] (95% CI), %	–	2.6 (–4.3 to 9.6)	7.2 (0.1 to 14.3)*	4.8 (–2.5 to 12.0)	–	4.4 (0.7 to 8.1)*	7.4 (3.8 to 11.0)***	8.8 (5.1 to 12.4)***
Reticulocyte hemoglobin								
Reticulocyte hemoglobin at baseline, mean (SD), pg	29.7 (2.0)	30.0 (1.9)	30.0 (1.9)	29.2 (2.8)	31.0 (1.5)	31.1 (1.5)	30.6 (1.8)	31.2 (2.1)
Reticulocyte hemoglobin at week 12, observed mean (SD), pg	30.5 (1.9)	30.8 (1.6)	30.8 (1.4)	30.6 (2.9)	31.1 (1.5)	31.7 (1.7)	31.6 (1.6)	32.0 (2.0)

	Baseline Hb <11 g/dL				Baseline Hb ≥11 g/dL			
	**Placebo**	**Ziltivekimab 7.5 mg**	**Ziltivekimab 15 mg**	**Ziltivekimab 30 mg**	**Placebo**	**Ziltivekimab 7.5 mg**	**Ziltivekimab 15 mg**	**Ziltivekimab 30 mg**
Change from baseline to week 12, estimated mean[a] (95% CI), pg, [*n*]	0.5 (–0.3 to 1.2) [*12*]	0.6 (–0.1 to 1.3) [*13*]	0.4 (–0.3, 1.2) [*11*]	1.1 (0.3 to 1.8) [*11*]	0.1 (–0.3 to 0.5) [*43*]	0.6 (0.2 to 1.0) [*39*]	0.8 (0.4 to 1.2) [*47*]	0.8 (0.4 to 1.2) [*44*]
Treatment difference[a] (95% CI), pg	–	0.1 (–0.8 to 1.1)	0.0 (–1.1 to 1.0)	0.6 (–0.4 to 1.6)	–	0.5 (0.0 to 1.0)	0.7 (0.2 to 1.2)**	0.7 (0.2 to 1.2)**

CI, confidence interval; Hb, hemoglobin; CKD, chronic kidney disease; TIBC, total iron-binding capacity; SD, standard deviation. n: Number of participants with change from baseline to week 12. *P<0.05, **P<0.01, ***P<0.001 versus placebo.

[a]Estimated from a mixed model for repeated measures with CKD stage (3a and 3b–5), concomitant iron medication, treatment group, visit, and treatment group-by-visit-by-subgroup as fixed factors, and baseline values as covariate.

Supplemental Table 3. Transferrin Saturation at Baseline and Week 12 and CRP at Baseline, by Baseline Transferring Saturation Quartile Groups, for Pooled Ziltivekimab Groups Versus Placebo

	**Placebo**				**Ziltivekimab (pooled)**			
**Transferrin saturation quartile groups**	**Quartile 1 ≤15.5% (*n*=19)**	**Quartile 2 >15.5% and ≤19.0% (*n*=15)**	**Quartile 3 >19.0% and ≤23.5% (*n*=19)**	**Quartile 4 >23.5% (*n*=13)**	**Quartile 1 ≤15.5% (*n*=49)**	**Quartile 2 >15.5% and ≤19.0% (*n*=56)**	**Quartile 3 >19.0% and ≤23.5% (*n*=44)**	**Quartile 4 >23.5% (*n*=49)**
Transferrin saturation								
Transferrin saturation at baseline, median (IQR), %	13.0 (9.5–14.5)	17.0 (16.0–18.5)	22.0 (21.0–22.5)	28.0 (26.0–37.0)	13.5 (11.0–15.0)	17.5 (16.8–18.5)	21.5 (20.5–22.5)	27.0 (25.5–32.0)
Transferrin saturation at week 12, median (IQR), %	14.5 (12.5–19.5)	16.0 (14.0–21.0)	20.0 (14.0–23.0)	23.5 (16.0–39.0)	21.5 (18.0–25.0)	22.0 (18.0–26.0)	26.5 (21.5–31.0)	31.0 (28.0–39.0)

	Placebo				Ziltivekimab (pooled)			
**Transferrin saturation quartile groups**	**Quartile 1 ≤15.5% (*n*=19)**	**Quartile 2 >15.5% and ≤19.0% (*n*=15)**	**Quartile 3 >19.0% and ≤23.5% (*n*=19)**	**Quartile 4 >23.5% (*n*=13)**	**Quartile 1 ≤15.5% (*n*=49)**	**Quartile 2 >15.5% and ≤19.0% (*n*=56)**	**Quartile 3 >19.0% and ≤23.5% (*n*=44)**	**Quartile 4 >23.5% (*n*=49)**
hsCRP								
hsCRP at baseline, median (IQR), %	10.6 (6.0–15.6)	5.6 (2.2–9.7)	4.6 (3.3–6.1)	4.5 (2.7–8.3)	7.3 (5.2–14.9)	6.3 (3.6–9.0)	4.9 (3.1–7.0)	4.6 (3.3–7.3)

hsCRP, high-sensitivity C-reactive protein, IQR, interquartile range.

Ziltivekimab data include the pooled 7.5 mg, 15 mg, and 30 mg doses. *n* values for the placebo group at week 12 were 16, 15, 16, and 10 for the ≤15.5%, >15.5% and ≤19.0%, >19.0% and ≤23.5%, and >23.5% transferrin saturation quartile groups, respectively. *n* values for the ziltivekimab group at week 12 were 42, 49, 40, and 45 for the ≤15.5%, >15.5% and ≤19.0%, >19.0% and ≤23.5%, and >23.5% transferrin saturation quartile groups, respectively.

Supplemental Table 4. Proportion of Participants With Baseline Hb <12/<13 g/dL or ≥12/≥13 g/dL by Sex

	**Baseline Hb < 12 for Women and <13 for Men**	**Baseline Hb ≥ 12 for Women and ≥ 13 for Men**
Women, *n* (%)	66 (51.2)	63 (48.8)
Men, *n* (%)	72 (53.3)	63 (46.7)

Hb, hemoglobin.

Supplemental Figure 1. Distribution of Baseline Hb by Sex

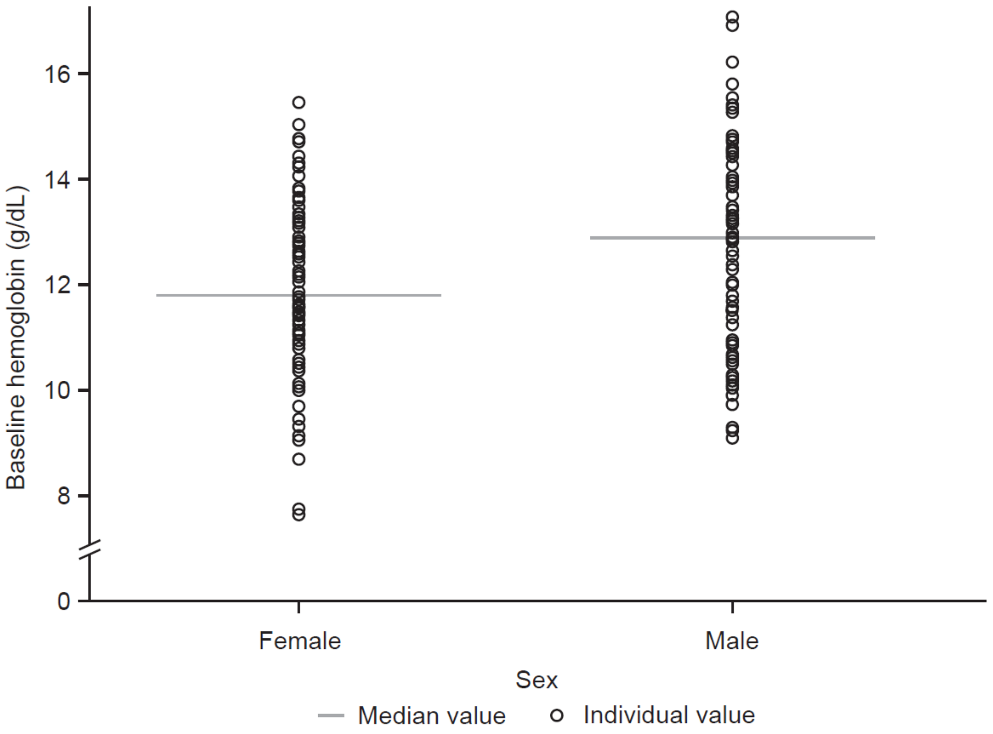

Hb, hemoglobin.
